# Teledermatology in medical education – a ‘rash’ decision?

**DOI:** 10.3402/meo.v20.30576

**Published:** 2015-12-15

**Authors:** Mariam Lami, Katherine Malabanan, Orlaith McAuliffe

**Affiliations:** Faculty of Medicine, Imperial College London, London, UK

The benefits of ‘teledermatology’ have been widely documented; most importantly, teledermatology has reduced referral times for patients saving money for the burdened NHS (1). However, with less need for face-to-face consultations (2), there may be fewer opportunities for medical students to get hands-on experience with dermatological conditions. While access to patient images will help enhance students' diagnostic abilities (3), may they be losing out on acquiring vital clinical skills, such as the art of building patient rapport? In this article, we intend to discuss the impacts of teledermatology, not on current or future patients, but on the students training to help them.

As a highly visual specialty, students will undoubtedly benefit from having access to a large store of dermatological images. Not only will students have exposure to what is common, they will also have the chance to see how rarer conditions present; this is an opportunity they may not have when practicing in a general dermatology clinic. Easy access to a wide variety of presentations of skin disease will also train the eye for spot diagnoses in a much quicker period of time. In one study comparing the eye movements of consultant dermatologists to registrars, consultants made quicker diagnoses. This is because consultants have more experience and therefore more advanced skills in eye pattern recognition (4). Therein lies the rub – there is ongoing uncertainty surrounding the use of patient images for educational purposes. Legalities regarding medical student access to the images needs to be finalized.

As previously alluded to, teledermatology creates doctors with a better base of knowledge. In the only published paper to have evaluated medical students' perspective on teledermatology, 88% agreed that it is beneficial for building knowledge and practice-based learning (3). However, communication skills and professionalism were felt to be neglected. Even with real-time videoconferencing, which has also been introduced to dermatological practice, communication with patients is limited and student learning can be minimal. An endless photo bank may create a doctor who can diagnose but not necessarily one who can empathize when dealing with the many associated psychosocial aspects within dermatology. William Osler once said ‘The good physician treats the disease; the great physician treats the patient who has the disease'. Could the integration of this tool lead students to become lazy in taking a holistic view of the patient?

Moreover, teledermatology may affect student skillsets. There have been issues with ‘zoning' when using teledermatology to diagnose patients, where more focus is placed on the main lesion while everything else remains in the periphery (5). Our generic history taking encourages us to build up an overall picture of factors that could affect patient health from family history to smoking. Another issue is the lack of physical contact with skin lesions. Often, examination with touch plays a key role in diagnosis and yet it is disregarded in this method which favors the use of two-dimensional images. Both zoning and lack of touch may compromise student clinical skills.

Because of a decreased need for consultations and a reduced workload for consultants and specialists, perhaps there will be more time for the holistic care of patients and for teaching and sharing of dermatological knowledge. From experience, we have spent time shadowing consultants with little opportunity to practice being doctors. The snowball effect of improved dermatological teaching may reach into primary and secondary care. However, could teledermatology trivialize dermatology in secondary care and create a loss of interest in the specialty? If so, we can expect a dramatic decline in job opportunities, with fewer specialists available to pass on their knowledge to the future generations. That being said, residents in Newark, USA who had no formal dermatological teaching, managed to improve their quiz scores through watching videostreamed lectures from Harvard University. (6). There is no longer an issue of space and time in sharing knowledge, thanks to online lectures being streamed worldwide.

Undeniably, teledermatology may benefit medical students with increased revision resources. But with fewer clinics to practice clinical skills and fewer dermatologists to pass on their knowledge, student education may rely solely on face-less two-dimensional images rather than real-life people. Further studies are needed to evaluate the impact of teledermatology on medical education. Is the use of teledermatology a ‘rash' decision for our current and future students? Only time will tell.

*Mariam Lami* Faculty of Medicine Imperial College London London, UK *Katherine Malabanan* Faculty of Medicine Imperial College London London, UK *Orlaith McAuliffe* Faculty of Medicine Imperial College London London, UK Email: om511@ic.ac.uk
